# Multiple endocrine neoplasias type 2B and RET proto-oncogene

**DOI:** 10.1186/1824-7288-38-9

**Published:** 2012-03-19

**Authors:** Giuseppe Martucciello, Margherita Lerone, Lara Bricco, Gian Paolo Tonini, Laura Lombardi, Carmine G Del Rossi, Sergio Bernasconi

**Affiliations:** 1 University of Genova, Associate Professor of Pediatric Surgery - DIPE, Via Gaslini, 5 Genova (16147), Italy; 2 Traslational Oncopathology National Cancer Research Institute, Genova (16100), Italy; 3 Laboratory of Molecular Genetic, Istituto G. Gaslini, Genova (16147), Italy; 4 Department of Pediatric Surgery, Ospedale Maggiore, Via Antonio Gramsci 14, Parma (43010), Italy; 5 Director Pediatric Department, University of Parma (43010), Italy

**Keywords:** Neurocristopathies, Neural Crest Cells, Cancer, MEN 2B, Multiple Endocrine Neoplasia, Medullary Thyroid Carcinoma, RET proto-oncogene, Thyroidectomy, Neuroblastoma, Hirschsprung's disease

## Abstract

Multiple Endocrine Neoplasia type 2B (MEN 2B) is an autosomal dominant complex oncologic neurocristopathy including medullary thyroid carcinoma, pheochromocytoma, gastrointestinal disorders, marphanoid face, and mucosal multiple ganglioneuromas. Medullary thyroid carcinoma is the major cause of mortality in MEN 2B syndrome, and it often appears during the first years of life. RET proto-oncogene germline activating mutations are causative for MEN 2B. The 95% of MEN 2B patients are associated with a point mutation in exon 16 (M918/T). A second point mutation at codon 883 has been found in 2%-3% of MEN 2B cases. RET proto-oncogene is also involved in different neoplastic and not neoplastic neurocristopathies. Other RET mutations cause MEN 2A syndrome, familial medullary thyroid carcinoma, or Hirschsprung's disease. RET gene expression is also involved in Neuroblastoma. The main diagnosis standards are the acetylcholinesterase study of rectal mucosa and the molecular analysis of RET. In our protocol the rectal biopsy is, therefore, the first approach. RET mutation detection offers the possibility to diagnose MEN 2B predisposition at a pre-clinical stage in familial cases, and to perform an early total prophylactic thyroidectomy. The surgical treatment of MEN 2B is total thyroidectomy with cervical limphadenectomy of the central compartment of the neck. When possible, this intervention should be performed with prophylactic aim before 1 year of age in patients with molecular genetic diagnosis. Recent advances into the mechanisms of RET proto-oncogene signaling and pathways of RET signal transduction in the development of MEN 2 and MTC will allow new treatment possibilities.

## 

Multiple Endocrine Neoplasia Type 2 B (MEN 2B) is a rare autosomal dominant complex neoplastic neurocristopathy characterized by the development of a number of tumors including medullary thyroid carcinoma (MTC) and pheochromocytoma (Pheo) with gastrointestinal symptoms, marphanoid facies and multiple ganglioneuromas (GN)/ganglioneurofibromas(GNf) [1]. MTC is present in 100% of MEN 2B cases, and it often already appears in the 1^st ^decade of life [2]. MTC is the main cause of death in MEN 2B patients who don't receive early or prophylactic treatment. Surgical treatment of MTC is the only effective therapy in cases with localized tumor.

Molecular analysis of the RET gene (**RE**arranged during **T**ransfection) has changed the history of this syndrome, as it allows the identification of MEN 2B mutations in asymptomatic patients, and let to perform prophylactic thyroidectomy in children. RET mutations can also be responsible for MEN 2A syndrome (MTC, PC and hyperparathyroidism) or familial MTC (FMTC). The same RET gene is causative for Hirschsprung's disease (HSCR) in a variable percentage of patients and HSCR can be associated with MEN 2 [3-8]. The molecular analysis has given an important contribution to understand Ret protein functions and the correlation between genotype and phenotype in RET mutations carriers.

According to genetic diagnosis, RET mutation analysis can provide early diagnosis and treatment of such a rare syndrome as MEN 2B, and becomes part of an international protocol responding these requirements.

### Neurocristopathies a unifying concept

Although the importance of the neural crest (NC) was first identified over a century ago, it has gained wide acceptance in vertebral development over the last 20 years [9]. The neural crest cells contribute to the formation of structures through-out the body. Therefore, 1 out 3 human congenital malformations involves structures related to NC.

Diseases arising from NC are particularly diverse in clinical presentation, including endocrinologic, cutaneous, neurological, digestive, or congenital syndromes [3,9,10].

Following these conditions, in 1974 Bolande suggested the name *neurocristopathies*. In his historical publication, the Author divided neural crest diseases into 2 basic forms [11,12]. The first includes the simple neurocristopathies, which are characterized by a single pathological process, generally unifocal and localized. The latter is represented by neurocristopathy syndromes and complex neurocristopathies that correspond to multifocal and varied associations of simple neurocristopathies (Table 1). Taking into account the Boland classification, Albinism, Hirschsprung's disease (HSCR), etc.. are considered simple non neoplastic neurocristopathies. Neuroblastoma and Medullary Thyroid carcinoma-only (MTC-only) are simple neoplastic neurocristopathies. Multiple Endocrine Neoplasia (MEN), Neurofibromatosis, and Familial Neuroblastoma associated with HSCR are complex neurocristopathies (see Table 1) [10-12].

**Table 1:** The Neurocristopathies Classification

***Simple neurocristopathies***	*Complex Neurocristopathies *
**Non Neoplastic-Dysgenetic**	**Neoplastic and Non-Neoplastic**
- Hirschsprung's disease	- Neurofibromatosis (Von Recklinghausen disease)
- Albinism	- Multiple endocrine neoplasia (MEN) type 1
- Mandibulofacial dysostosis	- MEN2A
- Otocephaly	- MEN2B
- Congenital Central Hypoventilation	- Neurocutaneous melanosis
- Syndrome	- Familial neuroblastoma with Hirschprung' s disease
**Neoplastic**	- CCHS + HSCR = Haddad syndrome
- Neuroblastoma	- Waardenburg + HSCR = Shah Waardenburg Syndrome
- Pheochromocytoma	
- Medullary thyroid carcinoma (MTC only)	
- Noncromaffin paraganglioma	
- Carcinoid tumors	

Since Multiple Endocrine Neoplasia (MEN) are complex neoplastic neurocristopathies, they are syndromes classified as type 1 and 2, each with specific phenotypic patterns [13,14]. MEN Type 1 is related to pituitary, parathyroid and paraneoplastic neuroendocrine tumours. MEN type 2 occurs in three clinical distinct varieties with MTC as the common manifestation (see Table 2). The three varieties are clinically distinct according to incidence, genetics, age of onset, associations with other diseases and prognosis [14]:

**Table 2:** Classification of MEN 2 and occurrence of MTC, and associated disorders (modified by Raue F et al, 2010

Subtype	MTC (%)	Pheo (%)	HPT (%)	Associated Diseases
MEN 2A	100	50	25	Cutaneus lichen amyloidosis, hirschsprung's Disease
**MEN 2B**	**100**	**50**	**-**	**Ganglioneuromatosis, marphanoid habitus, megacolon**
FMTC	95	-	-	Rare associated disorders

1) MEN 2A (Sipple's Syndrome) is characterized by MTC, pheochromocytoma (Pheo), and primary hyperplasia of the parathyroids (HPT).

2) MEN 2B is characterized by MTC, Pheo, mucosal ganglioneuromatos, and Marfanoid habitus. It is the rarest and most aggressive form of MEN type 2.

3) Familial MTC (FMTC) presents a low incidence of other associated disorders.

HPT is not a feature of MEN 2B. The MEN 2B has the highest mortality and morbidity. The high mortality reflects the early onset of MTC (generally during the first years of life). Unfortunately, at the time of the diagnosis an advanced MTC may be present.

### Clinical Features of MEN 2B

The clinical features have to be well known, especially in case of sporadic MEN 2B where they represent the only possibility to obtain a early suspect of the syndrome.

#### Gastrointestinal Manifestations

In most of the cases they are the first unspecific manifestations. The patients can present alvus disorders characterized by constipation alternating with diarrhea already in the first weeks of life. These types of troubles are getting worse. If the MEN 2B patient has a contrast enema X-ray, his/her colon may show reduced caliber without haustra; and some diverticula may be present in descending colon and sigma, other patients show a megacolon. Intestinal mucosa will progressively develop multiple pseudo-polyps as result of multiple ganglioneuromas (GN)/ganglioneurofibromas (GNf) [1]. Intestinal obstruction resulting from a colonic giant ganglioneuroma is rare [15,16].

#### Mucosal Multiple Ganglioneuromas (GN) and Ganglioneurofibromas (GNf)

Multiple mucosal pseudo-polyps and bumps become progressively evident in oral cavity, on the mucosal surface of the lips and on the tongue (Figure 1 and 2). They generally develop during the first months of life [17,18]. Every part of gastrointestinal tract is affected. The clinical examination of the oral cavity is very important for the early suspect of the syndrome. In every child with bumpy lips and tongue associated with intestinal constipation, MEN 2B should be suspected and excluded.

**Figure 1:**
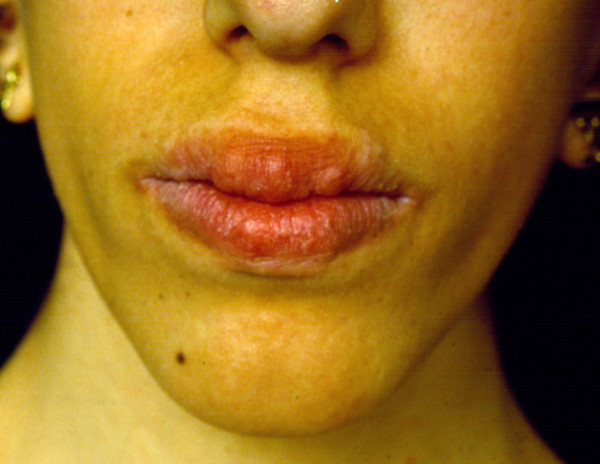
**Characteristic phenotype of MEN 2B including thickened lips with bumps**.

**Figure 2:**
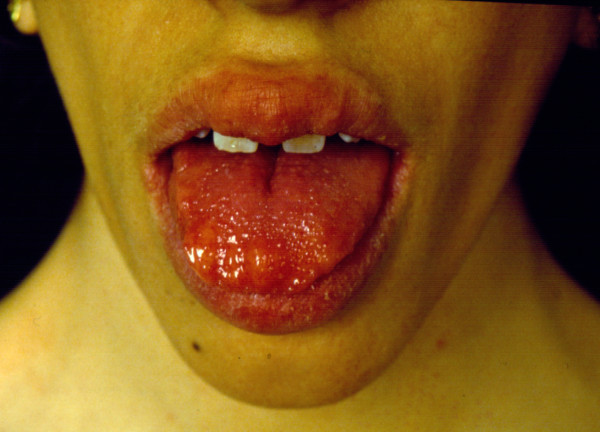
**Multiple pseudo-polyps and bumps on the tongue**. The lesions are mucous ganglioneurofibromas and ganglioneuromas.

GN and GNf lesions are characterized by tumors from mucosal and submucosal layers with enormous hypertrophy of nerve fibers (GNf) among ENS. Submucosal ganglion cells are usually present in normal numbers or organized in giant ganglia (GN) and always associated with large trunks of ENS nervous fibers. Ectopic ganglia inside lamina propia mucosae are present in most of the cases.

#### Marfanoid Habitus with Skeletal Deformations and joint Laxity

Marfanoid body habitus is presents in 65-75% of patients and it is characterized by an elongated face, large hand and feet and relatively long extremities [19-24].

The skeletal abnormalities are characterized by lordosis, kyphosis, kyphoscoliosis, joint laxity, talipes equinovarus and pectus deformity.

Although skeletal abnormalities may not be pronounced in the first few years, they can be considered one of the key to early diagnosis for the physician. A tall stature with disproportionately long limbs and digits, a long and narrow face with deep-set eyes, and a high, narrow palate are often combined with joint hypermobility and pectus deformities. Chest deformities such as pectus escavatum or carinatum are related to an overgrowth of the ribs, pushing the sternum outward or inward. Scoliosis is common in MEN 2B and the frequency is higher in adults. Untreated, significant spinal deformity can lead to chronic back pain and restrictive lung disease. There is a correlation between scoliosis and back pain, which occurs with greater frequency in adults with MEN2B than in the general population. Joint laxity can be pronounced in young children and may lead to delayed gross motor development. Joint dislocation is a rare occurrence. Mild contractures of elbows, knees, or toes are present in a small fraction of children and adults. The first toe is longer than the others and there is a wide space between the first and second toe (Figure 3). Adults often have an asthenic body habitus, and crowding of the teeth because of maxilla and mandible are narrowing.

**Figure 3:**
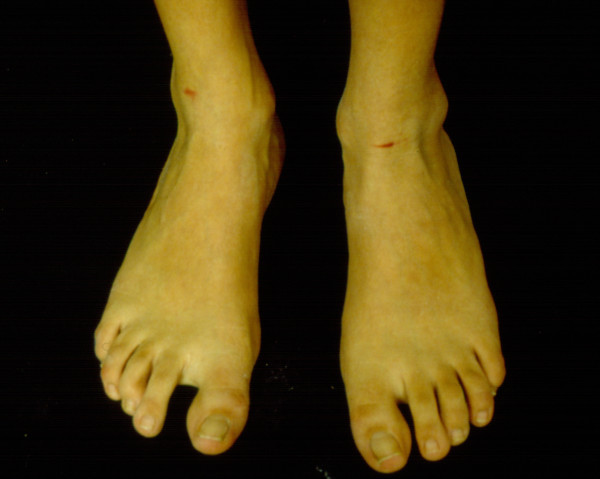
**The first toe is longer than the others and there is a wide space between the first and second toe**.

#### Inability to Cry Tears

In 2008, Brauckhoff et al reported that 86% of MEN 2B patients demonstrated inability to cry tears [25].

#### Palpable Cervical Tumor

Unfortunately, at time of diagnosis most of the sporadic MEN 2B patients present a palpable thyroid mass or thyroid nodules, all representing MTCs. As a matter of fact, MEN 2B is the least common (5-10% of all MEN), but the most aggressive form of MEN 2. Patients have a rapid onset of MTCs during the first year of life, and they have a more aggressive form of carcinoma with higher morbidity and mortality rates compared with patients with MEN 2A. Lymph node metastases are reported by the second year of life [4,26].

### Plasmatic Calcitonin and PGT

In all suspected MEN2B patients, plasmatic calcitonin (CT) in basal condition is measured. Values lower than 14 pg/ml and 19 pg/ml are considered normal in females and in males respectively [27]. Pentagastrin test (PGT) can be performed by infusion of a 0, 5 μg/kg of body weight of pentagastrin contained in 5 mL of 0, 9% NaCl as a bolus. Plasmatic CT is measured before the infusion of the bolus, and after 1, 5 and 5 minutes. Stimulated CT values are considered normal when lower than 30 pg/ml in females and 110 pg/ml in males [27,28].

PGT is handy used in older children or adult patients. Nevertheless it is not easy to perform this test in younger babies, because its normal range is not yet standardized in the first years of life and because during the first infancy it could not be completely without risks [4].

For these reasons the test is not very useful for early diagnosis.

### Intestinal Ganglioneuromatosis and Enzymo-histochemical Pre-operative Diagnosis of MEN 2B

Enzymo-histochemical studies of MEN 2B intestinal innervation have to be performed by the Acetylcolinesterase activity (AChE) technique, as described by Karnovsky and Roots [29,75,4]. AChE staining is very useful, because the picture is pathognomonic. The AChE is evaluated on rectal suction biopsies, as well as in the histochemical screening of HRSC.

The ganglioneuromas (GN) and the ganglioneurofibromas (GNf) are common conditions affecting peripheral nerves of MEN 2B intestinal wall. Their presence in the rectal mucosal and submucous layers is brown stained and AChE easily shows the enormous hypertrophy of nerve fibers (GNf) among ENS (Figure 4 and 5). Submucous ganglion cells are usually present in normal numbers or organized in giant ganglia (GN) and always associated with large trunks of ENS nervous fibers. Ectopic ganglia inside lamina propia mucosae are present in most of the cases. These peculiar AChE findings are so specific, that the diagnosis of MEN 2B is possible with the simple use of suction rectal biopsy.

**Figure 4:**
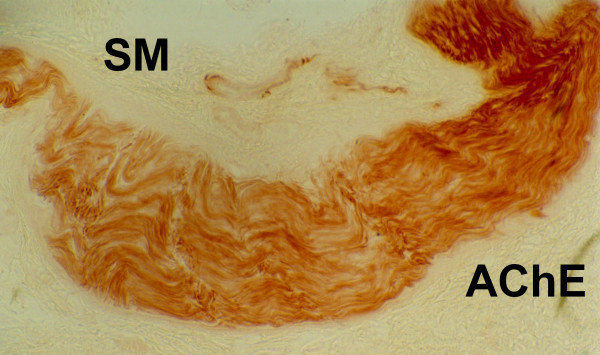
**Enzymo-histochemical studies of MEN 2B intestinal innervation performed by acetylcholinesterase on suction rectal biopsies: giant ganglioneurofibroma**. SM = submucous layer; AChE = acetylcholinesterase.

**Figure 5:**
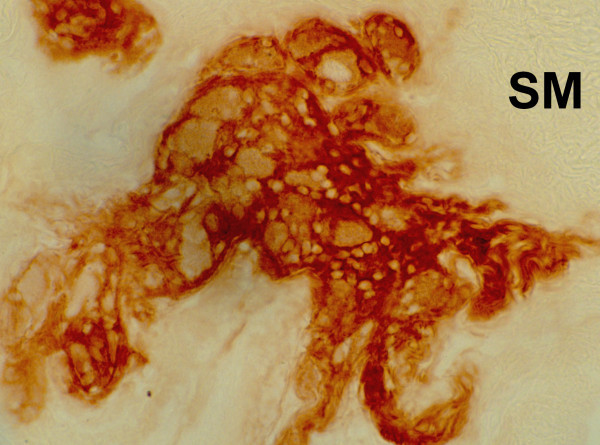
**Enzymo-histochemical studies of MEN 2B intestinal innervation performed by acetylcholinesterase on suction rectal biopsies: multiple ganglioneuromas and ganglioneurofibromas are brown stained**. SM = submucous layer.

### Genetic Analysis of RET Proto-oncogene in MEN type 2

According to literature data it is evident that MEN 2, unlike other inherited cancer syndromes associated with inactivation of tumor suppressor gene, results from activation or "gain-of-function" mutations of RET gene. A single activating mutation on one allele of the RET proto-oncogene is sufficient to induce neoplastic transformation [30].

Genetic analysis of the RET proto-oncogene, allows molecular diagnosis of different neurocristopathies. The analysis can be performed in MEN 2A and MEN 2B familial cases, in subjects presenting sporadic MTC, or affected by HSCR.

Activating mutations of RET seem to be of the order of 1: 500.000 in the general population [31]. Exons 10, 11, 13, 14, 15 and 16 of the RET proto-oncogene are analyzed for the detection of point mutations. In MEN 2A/FMTC cases, the analysis is firstly conducted in exons 10 and 11 by DGGE and subsequently in the remaining exons, if alterations had not been previously identified [4,8,32,33].

In 98% of classic MEN 2A families, germline mutations cluster to the extracellular cysteine-rich domain of the RET gene and involve single base pair substitutions in one of the five cysteine codons in exon 10 (609, 611, 618, 620) or 11 (634). The great majority of the mutation detected in MEN 2A are in codon 634, with C634R (Cysteine to arginine substitution). Rare mutations associated with MEN2A include those in exon 11 (insertion/duplications in codon 635, 637), 13 (codons 768, 790, 791), 14 (codon 804), and 15 (codon 891). The majority of FMTC kindreds also have germline mutations in the extracellular cysteine-rich region; however, in FMTC they are more evenly distributed among codon 618, 620 and 634. FMTC is also associated with non-cysteine mutations in the intracellular tyrosine kinase domain; this includes exon 13 (codons 768, 790, 791), 14 (codon 804) and 15 (codon 891). Recently it has been suggested that FMTC is part of MEN 2A spectrum, indicating variable allelic penetrance [30].

In MEN 2B patients, exon 16 is primarily screened since more than 95% of cases present the M918T mutation [34]. A second point mutation at codon 883 has been found in 2%-3% of individual with MEN 2B [35,36]. Tandem RET mutations of codons 805, 806 and 904 in *cis *configuration with the p.V804M mutation have also been reported in individuals with MEN 2B [37-39].

Finally, all HSCR patients were screened for mutations in exon 10, since some families presenting the association of HSCR and FMTC/MEN2A segregate one of the mutations affecting the cysteine residues in exon 10 [34].

DGGE analysis is performed as previously reported [40]. Sequencing of the altered PCR products is directly performed using dye terminator chemistry (Dye Terminator Cycle Sequencing Kit - ABI Prism Perkin Elmer, Norwalk, CT, USA) following the user's manual instructions. Electrophoresis of the cycle-sequencing products is carried out in an ABI 377 Automated Sequencer (Applied Biosystems, Foster City, CA, USA) and results are analyzed through an appropriate software.

In MEN 2B and FMTC are generally seen mutations involving intracellular non-cysteine codons in the tyrosine kinase domain. These mutations alter either adenosine triphosphate binding (codons 768, 790, 791, 804 and 891) or the substrate recognition pocket of the catalytic core (codon 918) resulting in altered substrate specificity of the RET protein. These altered RET isoforms cause aberrant phosphorylation of the substrate and activation of RET signaling pathways that induce cellular transformation [30].

### RET Mutations in Hirschsprung's Disease

Hirschusprung's disease is the commonest cause of intestinal obstruction in the early pediatric age group with a recognized recurrence risk of 4% for sibs of affected individuals in comparison with the general population incidence rate of 1/5000 live births with male to female ratio 4: 1. There is a remarkable association with other genetic diseases, including malformations and/or chromosomal anomalies that will be discussed successively in this chapter. Many genetic informations are obtained from animal models affected with colonic aganglionosis showing specific pattern of inheritance and genetic defects.

Sporadic occurrence accounts for 80-90% of HSCR cases with a variable expressivity (different length of aganglionic segments between patients), and an incomplete sex dependent penetrance (whether the individual shows a phenotype of his genotype or not). All these features of HSCR suggest a more complex pattern of inheritance (multifactorial) as well as the involvement of many genes (genetic heterogeneity)

Through segregation analysis on different sets of patients and their families, different forms of inheritance were suggested depending on the length of aganglionic segment. HSCR was classified as autosomal dominant with incomplete penetrance for long segment HSCR and autosomal recessive or multifactorial in short segments HSCR form [41,42].

A first step in understanding the molecular basis of HSCR was the observation of a little girl who was affected by total colonic aganglionosis with de-novo interstitial deletion in chromosome 10 (46xx, del.10 q11.21-q21.2) [43]. Further investigations allowed the reduction of this region into 200 kb which was the area of RET proto-oncogene [44]. The molecular strategy started with the published cDNA sequence at that time and the exon-intron structure of this gene was reconstructed by using a PCR based approach [45-47]. The intronic sequences flanking the 5' and 3' ends of each one of the first 20 exons (which were known at that time) were used to design primers and to amplify each exon. The analysis was later subjected to Single Strand Conformational Polymorphism (SSCP), leding to the identification of different forms of mutations [48].

RET gene was found to be mutated in about 35% of sporadic cases and 49% of familial HSCR cases and in a higher percent in long HSCR than in short type (76% vs 32%) [17,23]. RET mutations that lead to HSCR can occur throughout the 21 exons of the gene and at least 89 mutations have been identified, including nonsense mutations, missense mutations, small deletions, and insertions [49,50]. On the other hand, mutations leading to MEN2A or FMTC-familial medullary thyroid carcinoma are point mutations localized in one of 5 cysteins of the extracellular domains and they are activating mutations unlike those causing HSCR [45-47]. HSCR mutations in RET gene lead to loss of function alleles and they are heterozygous in nature which suggests haploinsufficiency.

About 5-10% of patients show other mutations in other genes as Glial cell Lined Derived Neurotrophic Factor (GDNF), Neurturin (NTN), Endothelin3 (EDN3), Endothelin B Receptor (EDNRB), Endothelin Converting Enzyme 1 (ECE1), The Transcriptional factor SOX10, Smad Interacting protein-1 (SIP1) and PHOX2B gene [42,50,54-56].

The small percentage of patients with known mutations rises the suspicion about the involvement of other modifier genes or additional risk factors, some of which being already mapped [53-55,57].

RET is primarly expressed during embryonic life in the neural crest, urogenital precursors, adrenal medulla and thyroid and later on throughout postnatal life in central and peripheral nervous systems and the endocrine system [49].

Mutations in RET gene play an essential role in two common neurocristopathies: MEN2 (OMIM 171400 and 162300) an autosomal dominant disorder caused by activating mutations and HSCR(OMIM 142623) which is believed to be caused by loss of function mutations.

Although HSCR and MEN2 are usually observed in isolated cases and probably they result from different molecular and cellular mechanisms due to different mutation types, the identification of RET mutations (C618 and C620) in families that have both HSCR and MEN 2 (FMTC and MEN2A) was surprising. The underlying mechanism that leads to both diseases is unknown [58] and it has been reported in a number of families who have HSCR but carry the mutation leading to MEN2 [51]. This may give clue about the peculiar molecular mechanisms of the previous diseases. In order to explain how the same mutations can produce such diverse phenotype, we may hypothesize that they are the results of mutations occurring in different periods of embryonic and postnatal life.

Another explanation for the complex inheritance pattern of HSCR and the low detection rate for RET mutations is the presence of several common polymorphisms of RET gene associated with HSCR causing variable risk. Specific RET haplotypes have been found to act as protective or predisposing factors or to modulate the severity of the disease [57,52,59,58-62]. A specific haplotype of a rare allele of Single Nucleotide Polymorphism (SNP) of exon 2 (A45A) has been strongly associated with HSCR while the haplotype of an allele in exon 14 SNP (S836s) has shown a low penetrant protective factor against the disease [62]. Another recent study supports the existence of low penetrant variant of the RET gene lying within or close to the ACA allele, which is believed to have an effect on either RET transcription, splicing or function, and considered a susceptible allele causing HSCR [63].

### Neuroblastoma and RET Proto-oncogene

The role of RET gene in neuroblastoma has been debated for several years. Indeed, RET is an essential gene for the development of NC the same tissue from which neuroblastoma origins. So, the possibility that RET gene is associated with neuroblastoma carcinogenesis has been investigated by several research groups. However, Hofstra et al and Peaston et al report no RET gene mutations in both sporadic and hereditary neuroblastoma; only one case of RET mutation associated with NME1 mutation has been reported by Leone et al [64-66]. It is interesting to note that no RET mutation has been observed in familial neuroblastoma although this malignancy onsets frequently in the first year of life indicating that the carcinogenesis process already starts during the embryonic life [67].

Experimental evidences indicate that abnormal RET gene expression may play a role in disturbing the physiological NC development and participate to the neuroblastoma cell formation. D'Alessio et al observed that RET expression induces neuroblastoma cells differentiation and more recently the same researchers demonstrated that TRKB oncogene, another gene involved in NC development, cooperate with RET to differentiate these cells [68,69]. Several neuroblastoma cell lines express RET together with other tyrosine kinase receptors of the GDNF family (GFR-1, -2 and -3). Bachetti et al show that several transcription factors deregulate RET expression in neuroblastoma and Kurotsuchi et al report that DOK family genes influence the RET gene activity in this tumor [70,71]. Finally, the role of RET in neuroblastoma seems to be strongly associated with the induction of neuroblastoma cell maturation by retinoic acid. Retinoic acid is a well known and potent inducer of terminal cell differentiation of neuroblastoma cells. Most of all neuroblastoma cell lines are sensitive to the retinoic acid activity and the acid has been also employed for the treatment of High-Risk neuroblastoma patients to induce terminal neuroblastoma cell differentiation after bone marrow depletion [72]. Angrisano et al have shown that several and complex events such as modification of DNA methylation are associated with RET activity by retinoic acid [73]. This observation should be taken in to account for the treatment of neuroblastoma cells by retinoic acid.

### Early Diagnosis and Prophylactic Surgery in MEN 2B

Prohylactic total thyroidectomy is performed in gene mutation carriers in accordance with their potential risk [84]. Genetic diagnostic screening for MEN 2A should include at least the cysteine-containing codons 10, 11, and 16, but also exon 13 and 14. It is now established that the risk groups are determined by the genotype and should be used to dictate timing of prophylactic surgery. Recommendations on the timing of prophylactic thyroidectomy and extent of surgery were presented at the International Multiple Endocrine Neoplasia Meeting in 2001. The risk was stratified into tree classes using genotype-phenotype correlations [17,18]. Children with codon 883, 918, and 922 mutations have to be classified as level 3 (MEN 2B), with the highest risk of early and aggressive MTC. Total thyroidectomy with central node dissection is recommended for patients with these mutations by the age of 6 months. Children with RET codon 611, 618, 620, and 634 mutations (MEN 2A) have to be classified as level 2 or as having a high risk of MTC. Thyroidectomy with or without central node dissection is recommended for patients with these mutations before the age of 5 years. Children with RET codon 609, 768, 790, 791, 804, and 891 mutations have to be classified as level 1 with the lowest risk of MTC. Operation in level 1 class is recommended at the age of 10 years [17].

In agreement with literature data, we believe that central compartment cervical lymphadenectomy should be performed during thyroidectomy for MEN 2B [26]. Homolateral lymph node exploration (2 compartments) has to be performed in cases with macroscopic evidence of carcinoma at surgery (Figure 6), and bilateral lymphadenectomy (3 compartments) in the presence of evident lymph node metastases. If mediastinal lymph nodes were metastatic according to CT-scan, limphadenectomy would be extended to the mediastinum (4 compartments) [74].

**Figure 6:**
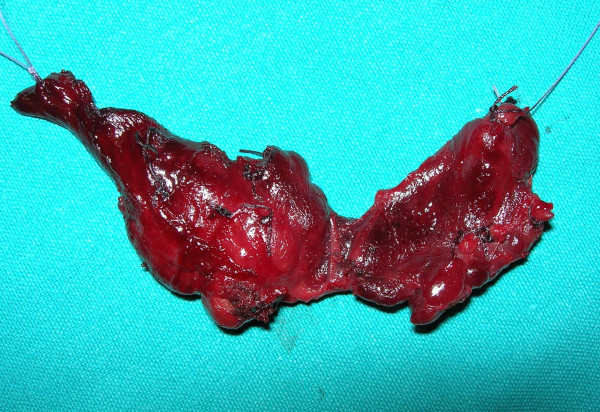
**Total thyroidectomy for a MEN 2B patient 3 years old**. Macroscopic evidence of carcinoma in the right lobe. Homolateral lymphadectomy together with central compartment lymphadectomy has to be performed during thyroidectomy.

### Immunohistochemical Post-operative Diagnosis in Resected Thyroids

Resected thyroids have to be weighed, measured, fixed in formalin and divided in three parts, namely right and left lobe, and hysthmus. Each part is divided by transverse serial sections into specimens, and embedded in toto. Histological sections are obtained from specimens embedded in paraffin, using the technique of semiserial sections. The sections obtained have to be stained alternatively with hematoxylin-eosin and histochemical reactions for tirocalcitonin (BioGenex, prediluited, policlonal) chromogranin A (BioGenex, prediluited, clone LK2H10) and tyroglobulin (BioGenex 1: 10, clone 2H11). For histochemical reactions, routine procedures for the antigen unmasking are used (treatment in microwave oven in citrate buffer, ph 6, 10 mM). Dako Envison Peroxidase is used as revelation system.

Patients underwent to total thyroidectomy show C cell hyperplasia with "in situ" MTC, or large nodules of MTC.

## Conclusions

Multiple Endocrine Neoplasia type 2B is a complex neoplastic neurocristopathy. MEN 2B is the rarest and most aggressive form of MEN. Prognosis in patients with MEN 2B syndrome depends on early diagnosis and surgical treatment. According to literature data, MTC occurs in 100% of MEN 2B and is very aggressive [2]. When it becomes clinically manifest, it can be too late for curative surgery. Metastases are present at surgery for clinical or biochemical evidence of MTC in 45% of MEN 2B patients [26].

In agreement with literature data, in our series of patients the first clinical signs of MEN 2B affected the gastroenteric system [32]. These symptoms are associated with typical marphanoid facies and multiple ganglioneuromas. The marphanoid features are not easy to identify in the first years of life, and ganglioneuromas at that time may be evident, but can be found if searched carefully. Gastrointestinal symptoms of MEN 2B generally include constipation or stipsis alternating with diarrhoea. These signs generally appear very early and sometimes are present already at birth, but they rarely suggest the diagnosis of MEN 2B. In children with constipation or stipsis alternating with diarrhoea, the presence of ganglioneuromas on the tongue and oral mucosa should be investigated, as well as the typical facies of MEN 2B and the family history of MTC or PC. In suspected cases, rectal biopsy has to be performed [29]. In both cases, the pathognomonic picture of MEN 2B was observed, namely: submucous plexus hyperplasia with giant ganglia (GN and GNf), submucous fibromatosis, and ectopic ganglia (Figure 4 and 5) [74]. In our opinion, rectal biopsy should be performed at first, as it allows diagnosis at an early disease stage. RET analysis is fundamental to confirm the diagnosis, and has to be extended to relatives. All carriers of MEN 2B mutations should undergo total thyroidectomy. On the basis of our experience and of literature data, prophylactic thyroidectomy is justified within the first year of life in patients with genetic diagnosis of MEN 2B [76,32,26,78].

The presentation of MEN 2B with thyroid mass can occur in cases with delayed diagnosis. In these patients the neuromas, the typical facies and the gastrointestinal symptoms are usually present. In these patients the diagnosis must be confirmed as soon as possible, with rectal biopsy and molecular analysis, in order to perform a total thyroidectomy associated with limphadenectomy. Fine needle aspiration of the mass is not advisable, in our opinion, because the result does not change the treatment, which anyway is based on surgery.

In the pre-operative work up we include cervical sonography and measurement of biochemical MTC markers: CT and CEA, useful for the follow up [27]. In case of thyroid mass, it is advisable to perform CT-scan, abdominal sonography and skeletal scintigraphy, in order to search for lymph node, hepatic or bone metastases.

Today, PGT has been replaced with molecular genetic analysis, which is much safer. Actually in pediatric patients PGT can be ill-tolerated and give false negative results. When positive, it can indicate the presence of carcinoma or C cell hyperplasia [79,27]. For these reasons, in our opinion this test is no longer indicated in the diagnosis of MEN 2B, whereas evaluation of basal plasma calcitonin CT in MEN 2B patients can play a role in their follow up.

In MEN 2B molecular genetic diagnosis, exon 16 is primarily screened since more than 95% of cases present the M918T mutation [4,32]. Finally, all HSCR patients were screened for mutations in exon 10, since some families presenting the association of HSCR and FMTC/MEN2A segregate one of the mutations affecting the cysteine residues in exon 10 [32]. A second point mutation at codon 883 has been found in 2%-3% of individual with MEN 2B [35,36]. Tandem RET mutations of codons 805, 806 and 904 in *cis *configuration with the p.V804M mutation have also been reported in individuals with MEN 2B [37-39].

After the genetic diagnosis of a patient affected with MEN 2B, every member of his/her family have to be screened for the M918T mutation. Even in the presence of family history of MEN 2B, genetic analysis should always be associated with enzymo-histochemical study on rectal biopsy, as it allows rapid diagnosis (1 day).

An interesting aspect is the association of MEN 2 with HSCR. It is well known that RET mutations can be causative for both HSCR and MEN 2 [80]. In particular, in MEN 2A patients the most frequent RET mutation (85%) affects codon 634 of exon 11, while in MEN 2B patients codon 918 of exon 16 is almost always involved. In HSCR, RET mutation can affect any portion of the gene. Interestingly, the most frequent mutations found in patients with the association of HSCR and MEN 2A/FMTC involve codons 609, 618 and 620 of exon 10 [82,83,33,45].

In HSCR patients, molecular analysis of standard MEN 2A/FMTC mutations is therefore recommended to identify a subpopulation of patients carrying mutations with potential oncologic risk.

The Ret protein is a tyrosine kinase receptor, that plays an important role in the activation of signalling pathways, through the phosphorylation of key tyrosine residues, in response to different ligands. In MEN 2A and MEN 2B, gain of function RET mutations result in the constitutive activation of the tyrosine kinase receptor, with subsequent phosphorylation and overtrasmission of the signal by different downstream pathways. The latter can be specifically activated by the different mutations, which therefore result in a large spectrum of possible phenotypes (MEN 2A, MEN 2B, FMTC, with different degrees of penetrance and expressivity). On the contrary, RET inactivating mutations are associated with HSCR. Loss of function mutations result in a reduction of the amount of functional Ret protein on the cell surface. Mutations found in patients with HSCR and MEN 2 association are able to activate the signalling pathways, like in isolated MEN 2, but the mutated isoform is unable to translocate to the cell surface. The result of activation in the thyroid and adrenal glands is tumorigenesis, while the decrease of functional protein on the cell surface causes HSCR phenotype [34].

In agreement with literature data, we believe that central compartment cervical lymphadenectomy should be performed during thyroidectomy for MEN 2B [26]. Homolateral lymph node exploration (2 compartments) has to be performed in cases with macroscopic evidence of carcinoma at surgery, and bilateral lymphadenectomy (3 compartments) is necessary in the presence of evident lymph node metastases. If mediastinal lymph nodes are metastatic according to CT-scan, limphadenectomy has to be extended to the mediastinum (4 compartments) [81].

Despite autotransplantation of parathyroid glands in the forearm is usually performed, in pediatric patients we prefer to preserve them in their primary site, in order to avoid traumas and mechanical insults, that are frequent in childhood upper limbs [76,26].

MEN 2B patients have to be followed year by year with measurement of CEA and CT, markers of possible MTC relapse (more strictly in the first year after surgery). While urinary metanephrines and fractionated catecholamines (epinephrine, norepinephrine, dopamine) are useful to identify possible development of PC.

In conclusion, early diagnosis and treatment of patients with MEN 2B are essential to their survival. The rarity of this syndrome can cause delayed diagnosis. MEN 2B is characterized by early clinical signs as nonspecific alvus disorders and, later, development of the typical facies and presence of ganglioneuromas. These signs, that precede tumor development, should suggest the diagnosis, which is based on rectal biopsy and genetic analysis. The protocol and diagnostic algorithm of MEN 2B that we propose (see Additional file 1) seems to offer the best life expectancy to patients affected by MEN 2B syndrome [84]. Moreover, recent advances into the mechanisms of RET proto-oncogene signaling and pathways of RET signal transduction in the development of MEN 2 and MTC will allow new treatment possibilities.

## Competing interests

The authors declare that they have no competing interests.

## Authors' contributions

MG carried out clinical, histochemistry studies and surgical activity, and participated in the design and coordination of the study. LM carried out the genetic studies and drafted the manuscript. LB carried out the genetic studies and drafted the manuscript. GPT carried out the genetic studies. LL worked clinical studies. CGDR worked clinical studies. SB worked clinical studies and participated in the design of the study.

All authors read and approved the final manuscript.

## Supplementary Material

Additional file 1 **Additional file 1**. Algorithm for diagnosis and treatment of MEN 2B. GI = gastrointestinal symptoms. Click here for file

## Aknowledgement

Authors thank Silvia De Luca for editing the manuscript.
