# Supplementary figures and images for: Multiple endocrine neoplasias type 2B and RET proto-oncogene
Source: Ital J Pediatr. 2012 Mar 19;38:9. doi: 10.1186/1824-7288-38-9 (PMC3368781; doi:10.1186/1824-7288-38-9)

Additional file 1. Algorithm for diagnosis and treatment of MEN 2B. GI = gastrointestinal symptoms

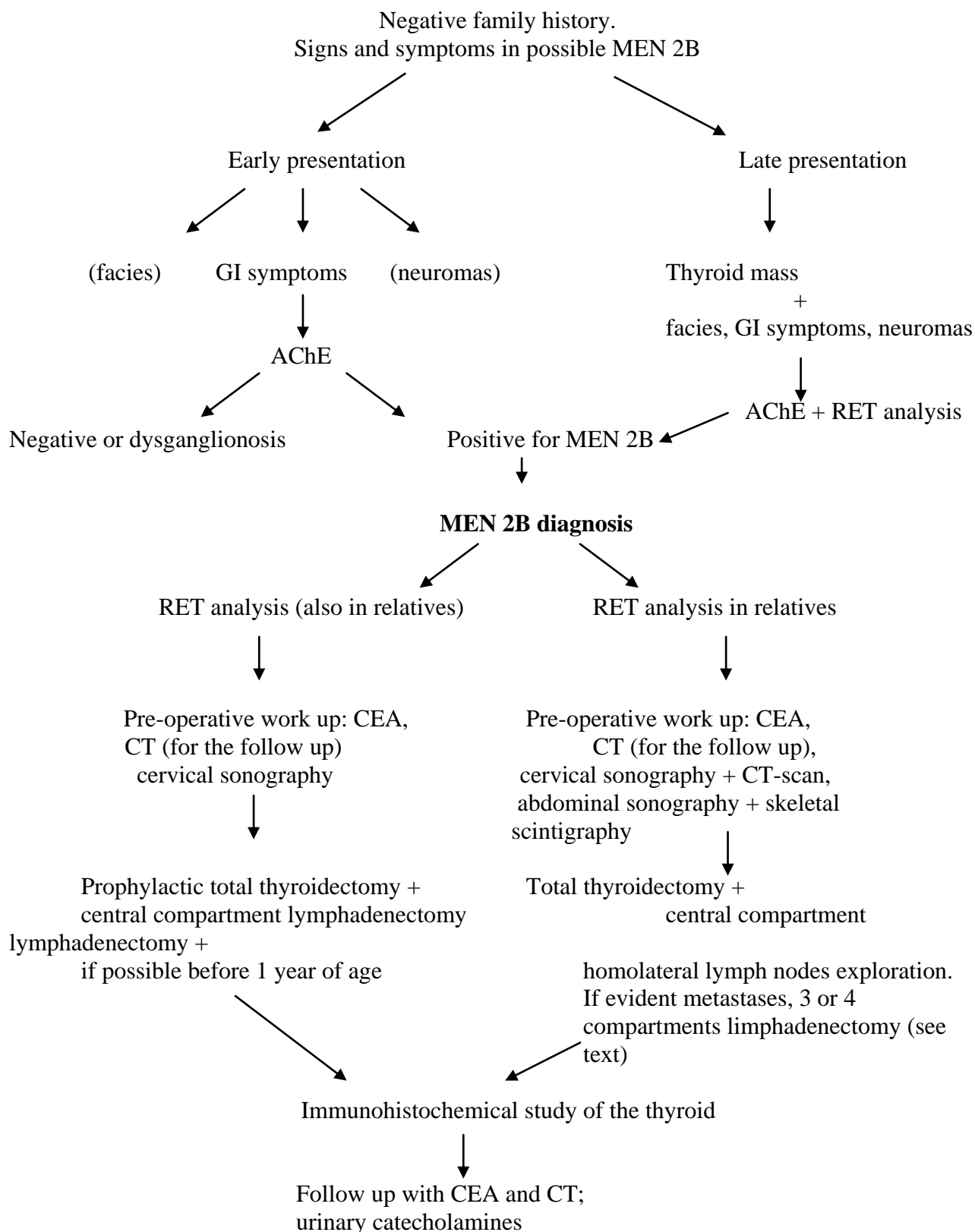

Supplement: Additional file 1 — Algorithm for diagnosis and treatment of MEN 2B. GI = gastrointestinal symptoms. [file 1824-7288-38-9-S1.PDF]
